# Flea-Borne Rickettsioses and Rickettsiae

**DOI:** 10.4269/ajtmh.16-0537

**Published:** 2017-01-11

**Authors:** Lucas S. Blanton, David H. Walker

**Affiliations:** 1 Division of Infectious Diseases, Department of Internal Medicine, University of Texas Medical Branch, Galveston, Texas.; 2 Department of Pathology, University of Texas Medical Branch, Galveston, Texas.

## Abstract

*Rickettsia typhi* and *Rickettsia felis* are flea-borne rickettsiae that are distributed throughout the world. This mini-review outlines the ecology and epidemiology of flea-borne rickettsioses; highlights important clinical, diagnostic, and therapeutic considerations; and discusses areas of uncertainty regarding *Rickettsia felis* and other rickettsiae harbored by fleas.

## Introduction

Members of the genus *Rickettsia* are obligately intracellular Gram-negative organisms that are transmitted to humans via hemophagous arthropod vectors. The rickettsiae have traditionally been classified as belonging to either the spotted fever or typhus group.^1^ *Rickettsia typhi* and *Rickettsia felis* are flea-borne pathogens, which both cause acute undifferentiated febrile illness throughout the world. *Rickettsia typhi* is a member of the typhus group and is the agent of murine typhus (endemic typhus).^2^ *Rickettsia felis* causes a similar illness, and although disease is often referred to as flea-borne spotted fever,^3^ the organism is a member of the transitional group, which consists of rickettsiae residing in a phylogenetic clade between the spotted fever and typhus groups and possessing phenotypic features that are difficult to classify.^1^ This mini-review outlines the ecology and epidemiology of flea-borne rickettsioses, highlights important clinical features, and discusses areas of uncertainty regarding *R. felis* and other rickettsiae harbored by fleas.

## Epidemiology

Murine typhus is distributed along warm coastal regions throughout the world, where the primary mammalian reservoir (*Rattus* spp.) and flea vector (*Xenopsylla cheopis*) thrive. *Rickettsia typhi* is acquired by fleas while feeding on rickettsemic rats. The organism infects the midgut epithelium of the flea and is shed in the feces, where it is transmitted to humans by the inoculation of *R. typhi*-laden flea feces onto flea bite wounds or mucous membranes.^4^ Murine typhus is often indistinguishable from other causes of fever in the tropics and is likely vastly under diagnosed. Indeed, it is increasingly recognized in returning travelers^5^—at times with severe manifestations.^6^,^7^

In 1944, there were 5,401 cases of murine typhus reported in the United States. In the following years, with the implementation of rat and vector control programs employing rodent trapping and dichlorodiphenyltrichloroethane (DDT), the number of cases dropped precipitously. By 1956, fewer than 100 cases were reported in the United States.^8^ The remaining endemic foci of murine typhus are in southern California and south Texas, where there exists an apparent alternate cycle of transmission for *R. typhi* involving opossums and cat fleas (*Ctenocephalides felis*).^9^,^10^ Because this mode of transmission occurs at the ecological overlap of opossums and humans, it is often referred to as the suburban cycle of transmission. This is juxtaposed to the urban cycle of transmission involving rats, which generally occurs in metropolitan areas. In Texas, the suburban cycle seems to be associated with the emergence of murine typhus in communities extending beyond the endemic range established in the last several decades.^11^,^12^ Domestic cats and dogs are often parasitized by *C. felis*, and previous reports have speculated cats as sources of local outbreaks.^13^

Since the discovery of *R. felis* in a laboratory colony of *C. felis* in 1990,^14^ the agent has been found to infect a variety of other arthropods.^15^ Since the mid-1990s, it has been increasingly recognized as a cause of human infection throughout the world.^3^ The primary reservoir and vector of *R. felis* is thought to be *C. felis*, where it is vertically transmitted to progeny (for up to 12 generations) despite the absence of an infectious blood meal.^16^ A mammalian reservoir host has yet to be elucidated. Cofeeding experiments have demonstrated the transmission of *R. felis* from infected to naïve fleas feeding on mice. The organism was detected within the skin of these mice, which suggests bites of infected fleas as a mode of human transmission.^17^

## Clinical Features, Diagnosis, and Treatment

Murine typhus is characterized by the abrupt onset of fever with accompanying headache, chills, myalgia, and malaise.^18^ Rash, which is the sign that often prompts a clinician to consider a rickettsiosis, is absent in 50%^2^ and may be present in as few as 20% of those with darkly pigmented skin.^19^ When present, rash is usually macular or maculopapular with a truncal distribution. Murine typhus is often accompanied by gastrointestinal symptoms (i.e., anorexia, nausea, vomiting, and abdominal pain), and in severe cases may be complicated by pulmonary, renal, and neurologic dysfunction. Death is reported in 4% of hospitalized patients.^18^ The clinical features of *R. felis* infection are similar to murine typhus—description is based on a growing number of case reports in the last two decades as summarized in a review by Parola.^3^ Notable differences include rash in 75% and eschar in 13% of these reported cases. An eschar-like lesion has only been described once in a patient with murine typhus.^20^ To our knowledge, there has never been a death attributed to infection with *R. felis*.

Isolation of the organism is definitive evidence of infection, but because of the need for technical expertise and biosafety level 3 containment, it is rarely performed. Interestingly, to our knowledge, *R. felis* has never been isolated from a clinical sample—infection has been confirmed by polymerase chain reaction (PCR) and sequencing. The immunohistochemical demonstration of rickettsiae within skin biopsy or tissue specimens is effective, but like culture, it is not available to most clinicians, and *R. felis* has not been visualized in human lesions. Serology continues to be the mainstay of diagnosis, but as demonstrated in patients with murine typhus, antirickettsial antibodies are present in less than 20% at 7 days of illness.^21^ Therefore, serodiagnosis requires both acute- and convalescent-phase sera to demonstrate seroconversion or a 4-fold titer increase. The serologic method of choice is the immunofluorescence assay. Serologically, anti-*R. felis* antibodies are cross reactive with spotted fever group antigen (e.g., *R. rickettsii* and *R. conorii*) and variably cross reactive with typhus group antigen.^22^–^25^ Unfortunately, as discussed below, peripheral blood PCR is not sensitive for the detection of rickettsemia for rickettsioses other than *R. felis*.

Doxycycline is the treatment of choice for all rickettsioses. Fever usually abates within 48–72 hours after initiation. A 7-day course is the usual duration of treatment.^2^ Chloramphenicol is an alternative drug but is likely less effective. In the United States, the oral formulation of chloramphenicol is not available, and the intravenous formulation is extremely difficult to acquire. Fluoroquinolones (e.g., ciprofloxacin and levofloxacin) are also alternatives, but as the case with chloramphenicol, they are associated with longer times to defervescence than doxycycline when used for murine typhus (4.23 days for ciprofloxacin, 4.00 days for chloramphenicol, 2.89 days for doxycycline).^26^

## *Rickettsia felis*: Unresolved Issues

The advent of sensitive molecular techniques has improved the diagnostic capability for many infectious diseases, but for rickettsioses, detection of circulating organisms by PCR has proved disappointing, with sensitivity of detection in peripheral blood dependent on the degree of infected endothelial cell destruction (i.e., severe or fatal cases).^27^,^28^ The detection of *R. felis* in humans seems to defy this principle. The organism has been detected in the blood and cerebrospinal fluid of those with an alternative and more compelling diagnosis (malaria, cryptococcal meningitis, and scrub typhus).^29^ In the past several years, there have been increasing reports of *R. felis* as a cause of febrile illness in Africa. Two independent groups, working in Kenya and Senegal, described molecular evidence of *R. felis* within the blood of 3.7%^30^ and 4.4%^31^ of febrile subjects, respectively. Later studies, incorporating the use of controls, have revealed the detection of *R. felis* DNA within both febrile and afebrile subjects. In western Kenya, 7.2% of febrile patients were found to have *R. felis* versus 3.4% of controls (odds ratio 2.20, 95% confidence interval: 1.03–4.70, *P* = 0.04).^32^ In Gabon, investigators found evidence of *R. felis* in 10.2% of febrile subjects versus 3.3% of afebrile subjects—a difference deemed statistically nonsignificant.^33^ In both these studies, it is unclear if other frequent causes of febrile illness were excluded. In the former study, *Plasmodium* spp. were detected in 79% of febrile patients with molecular evidence of circulating *R. felis* DNA and 73% of febrile subjects without amplification of rickettsial DNA.^32^ A multisite international study of febrile patients found a higher prevalence of *R. felis* PCR positivity in the malaria-endemic countries of Senegal (15%), Gabon (10%), and Mali (3%) versus Algeria (1%), Morocco (2%), Tunisia (0%), and France (0%) (*P* < 0.001). As in the aforementioned study by Maina and others, a large proportion (24%) of febrile subjects were not only positive for *R. felis* but were found to have circulating *Plasmodium* species. Interestingly, a group of afebrile controls in Senegal were positive for *R. felis* (4%) compared with none of the afebrile controls who resided in France.^34^

*Rickettsia felis* DNA has also been detected from skin lesions as well as from intact skin. A case report of a febrile Senegalese baby with vesicles and ulcerations (cutaneous findings atypical for a rickettsiosis) attributed her illness to *R. felis*, as the nucleic acid of the organism was detected from a lesional swab. Unlike a typical rickettsial infection, the child failed to develop antirickettsial antibodies in the weeks following her illness^35^—a curious phenomenon with speculative hypothetic explanations noted also in other reports.^29^,^34^ Another study performed in Senegal, which examined pathogens collected from eschars, detected *R. felis* DNA in 7.4% of tested lesions, but *R. felis* DNA was also detected from the intact skin of asymptomatic Senegalese volunteers. Swabs collected from the skin of healthy controls in France were negative.^36^

In addition to *R. felis*–infected *C. felis*, the organism's DNA has been detected in a variety of other fleas, ticks, mites, and lice, as summarized in a review by Brown.^37^ It has also been detected molecularly in both *Anopheles*^38^ and *Aedes*^39^ mosquitoes in Africa. Further characterization has been carried out experimentally in *Anopheles gambiae*, in which *R. felis* was acquired through a very heavily infected artificial blood meal, disseminated to various mosquito tissues, and was transmitted to mice through mosquito feeding.^40^ Although these experiments demonstrated the potential vector competence of *An. gambiae*, the role that infected mosquitos play in the epidemiology of *R. felis* infections in humans is unknown. In addition to these aforementioned blood-feeding arthropods, *R. felis* has also been cultured from the nonhematophagous book louse (*Liposcelis bostrychophila*),^41^ where its genetic diversity, compared with isolates obtained from other arthropods, is surprisingly great.^42^ Finally, *R. felis* DNA has even been detected in ape feces^43^—the implications of this finding are yet to be elucidated.

The presence of *R. felis* DNA circulating within healthy volunteers, its presence in those with parasitemia (*Plasmodium* spp.), and the absence of antirickettsial antibodies to support an immune reaction to the presence of *R. felis* are confounders that greatly limit the definitive proof of the causality of *R. felis* as the etiology of fever in Africa. The hypothesis regarding the transmission of *R. felis* by the bites of infected mosquitoes is compelling but does not reconcile the lack of an antibody response, which is inconsistent with the conventional wisdom regarding infection with pathogenic rickettsiae. Another attractive hypothesis attributes the amplification of *R. felis* DNA to the contamination of human skin with *R. felis*–infected booklice, which are often ubiquitous in the environment.^44^

## Other Flea-Borne Rickettsiae

The search for rickettsiae within fleas has revealed evidence of organisms which appear closely related to *R. felis*. These *R. felis*–like organisms have been discovered by PCR from *Ctenocephalides* spp. collected from cats and dogs on the Myanmar—Thai border,^45^ *C. felis* collected from animals killed on South Carolina highways,^46^ and *X. cheopis* collected from rats in Egypt.^47^ As with *R. felis*, these organisms seem to be distributed throughout the world. In a study of fleas collected from the Asembo District of western Kenya, genomic analysis and bacterial isolation revealed the presence of a rickettsial organism within a variety of fleas (i.e., *C. felis*, *Ctenocephalides canis*, *Pulex irritans*, and *X*. *cheopis*). This organism is closely related to *R. felis* but has enough genetic heterogeneity for proposal of a new species designation—*Candidatus* Rickettsia asemboensis.^48^–^50^ In addition to other sites in Africa,^51^ similar sequences obtained from *C. felis* collected in Israel,^52^ Ecuador,^53^ Colombia,^54^ and California^55^ reveal the widespread distribution of this organism. Another *R. felis*-like agent, *Candidatus* Rickettsia senegalensis, has been isolated from *C. felis* collected in Senegal.^56^ It has also been found within *C. felis* in California^55^ and Texas.^57^ Currently, little is known regarding the role of these *R. felis*-like organisms in human disease. Their pathogenic potential, as demonstrated in humans or in animal models, has not been described. Furthermore, nothing is known of their role in modulating the vertical and horizontal transmission of other sympatric rickettsiae.

## Conclusions

*Rickettsia typhi* and *R. felis* cause flea-borne rickettsioses, which are important but underrecognized causes of fever throughout the world. As undifferentiated febrile illnesses mimic a variety of other infectious diseases, awareness by clinicians is paramount to the initiation of appropriate therapy. Despite several reports implicating *R. felis* as a prevalent cause of fever in Africa, and despite the growing knowledge of newly discovered rickettsiae-infecting fleas, there is much to be learned regarding the ecology and pathogenicity of these organisms.
